# The X-Ray Crystal Structure of *Escherichia coli* Succinic Semialdehyde Dehydrogenase; Structural Insights into NADP^+^/Enzyme Interactions

**DOI:** 10.1371/journal.pone.0009280

**Published:** 2010-02-18

**Authors:** Christopher G. Langendorf, Trevor L. G. Key, Gustavo Fenalti, Wan-Ting Kan, Ashley M. Buckle, Tom Caradoc-Davies, Kellie L. Tuck, Ruby H. P. Law, James C. Whisstock

**Affiliations:** 1 Department of Biochemistry and Molecular Biology, Monash University, Clayton Campus, Melbourne, Victoria, Australia; 2 School of Chemistry, Monash University, Clayton Campus, Melbourne, Victoria, Australia; 3 Department of Molecular Biology, The Scripps Research Institute, La Jolla, California, United States of America; 4 ARC Centre of Excellence in Structural and Functional Microbial Genomics, Monash University, Clayton, Melbourne, Victoria, Australia; 5 Australian Synchrotron, Clayton, Victoria, Australia; University of Canterbury, New Zealand

## Abstract

**Background:**

In mammals succinic semialdehyde dehydrogenase (SSADH) plays an essential role in the metabolism of the inhibitory neurotransmitter γ-aminobutyric acid (GABA) to succinic acid (SA). Deficiency of SSADH in humans results in elevated levels of GABA and γ-Hydroxybutyric acid (GHB), which leads to psychomotor retardation, muscular hypotonia, non-progressive ataxia and seizures. *In Escherichia coli*, two genetically distinct forms of SSADHs had been described that are essential for preventing accumulation of toxic levels of succinic semialdehyde (SSA) in cells.

**Methodology/Principal Findings:**

Here we structurally characterise SSADH encoded by the *E coli gabD* gene by X-ray crystallographic studies and compare these data with the structure of human SSADH. In the *E. coli* SSADH structure, electron density for the complete NADP^+^ cofactor in the binding sites is clearly evident; these data in particular revealing how the nicotinamide ring of the cofactor is positioned in each active site.

**Conclusions/Significance:**

Our structural data suggest that a deletion of three amino acids in *E. coli* SSADH permits this enzyme to use NADP^+^, whereas in contrast the human enzyme utilises NAD^+^. Furthermore, the structure of *E. coli* SSADH gives additional insight into human mutations that result in disease.

## Introduction

**Figure 1:**
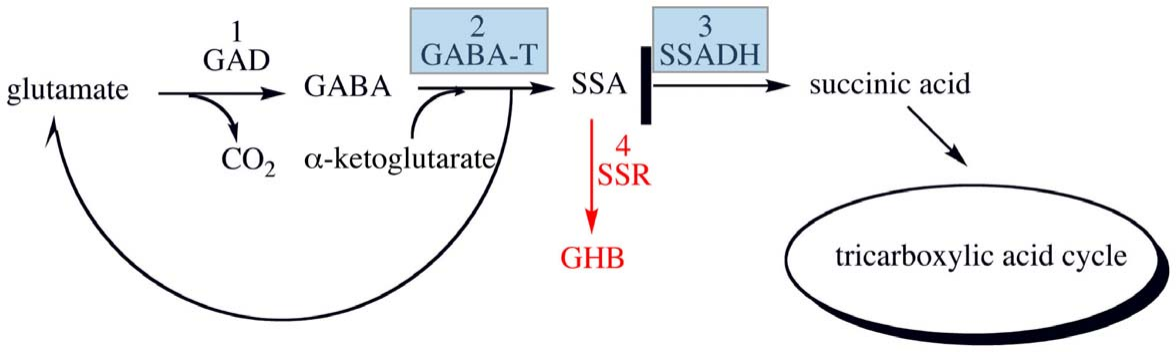
Enzymes and metabolites involved in the GABA shunt. Enzymes catalysing reactions are numbered, (1) glutamate decarboxylase (GAD), (2) γ-aminobutyric transaminase (GABA-T), (3) succinic semialdehyde dehydrogenase (SSADH) and (4) succinic semialdehyde reductase (SSR). The blue highlighted boxes show enzymes that are found in the *gab* operon of *E. coli*. The thick black line indicates the pathway is blocked, SSA is then converted to γ-hydroxybutyrate (GHB) by succinic semialdehyde reductase (SSR), pathway coloured in red, which is described in SSDAH deficiency. Figure adapted from Blasi *et al*. [9]

Succinic semialdehyde dehydrogenase (SSADH) belongs to the aldehyde dehydrogenases (ALDH) superfamily [1] and has been identified and purified from mammals [2], [3], [4], [5] as well as from microorganisms [6], [7], [8]. SSADH plays a key role in mammalian neurobiology, where it functions in the metabolic pathway termed the “γ-aminobutyric acid (GABA) shunt” in the brain. In the GABA shunt, the inhibitory neurotransmitter GABA is synthesised from glutamic acid by glutamic acid decarboxylase (GAD) [9], [10]. GABA is then metabolised in a two-step reaction. First, GABA-transaminase (EC 2.6.1.19) catalyses the breakdown of GABA in the presence of α-ketoglutarate to produce succinic semialdehyde (SSA) and glutamic acid (Figure 1). SSA is then converted to succinic acid (SA) by the NAD^+^/NADP^+^-dependant enzyme succinic semialdehyde dehydrogenase (SSADH; EC 1.2.1.24) [11]. Hence, GABA is channelled into the tricarboxylic acid cycle in the form of SA. Alternatively, SSA can be converted to γ-hydroxybutyric acid (GHB) by succinic semialdehyde reductase [12] (see Figure 1).

Autosomal deficiency of SSADH results [13], [14] in serious disease, with patients displaying varying degrees of psychomotor retardation, muscular hypotonia, non-progressive ataxia and seizures [15], [16]. As a result of a failure to properly metabolise SSA, SSADH deficiency leads to an accumulation of GABA, SSA and GHB (Figure 1). Accordingly, patients exhibit a ∼230 fold [17], [18] increase in levels of cerebrospinal fluid GHB as well as a modest 3-fold increase in GABA levels [16], [17], [19], [20]. The increase in GABA, SSA as well as GHB levels are all thought to contribute to SSADH deficiency disease through a complex range of signalling and developmental effects (for a comprehensive review see Knerr *et.al*.[18]).

In *Escherichia coli* [21], like in mammals, SSA can cause oxidative damage and two SSADH genes, the *gabD* and *sad* (also called *yneI*) genes, have been identified [21]. The *gabD* gene encodes a NADP^+^ dependent SSADH (EC 1.2.1.24) and is located in the *gab* operon. The products of the gab operon (which comprises *gabT* (γ-aminobutyrate transferase), *gabD* (SSADH), *gabP* (GABA permease) and *gabC* (a regulatory gene) [22]) drive GABA catabolism and permit cells to utilise GABA as the sole nitrogen source [23], [24]. The *sad* gene encodes for a NAD^+^ dependent SSADH (EC 1.2.1.16 and shares 32% identity with *gabD*) and is an orphan gene[25]. The *sad* gene is induced by exposure to exogenous SSA and functions primarily to prevent its accumulation in the cell. Furthermore, the *sad* gene product may also enable growth on putrescine as the nitrogen source [25].

Recently, the structure of human SSADH has been published [26]. These data suggest that a redox switch mediated via a reversible disulfide bond (between Cys340 and Cys342) in the catalytic loop regulate human SSADH activity such that formation of the disulfide bond results in the catalytic loop adopting a closed conformation that blocks access to the substrate and cofactor binding sites. Reduction of the disulfide bond leads to a large structural change where the catalytic loop switches to an open conformation permitting access to the substrate and cofactor binding sites (r.m.s.d. 4.1 Å over 11 residues of the catalytic loop). Shortly after the human SSADH structure was published, the structure of SSADH from *Burkholderia pseudomallei*, without a substrate or cofactor (PDB ID: 3ifg and 3ifh), was deposited into the PDB by the Seattle Structural Genomic Centre for Infectious Disease.

Despite these recent studies, the structural information of SSADH and its interactions with cofactor remains scarce. Here we report a 2.3 Å X-ray crystal structure of the *gabD* gene product (NADP^+^-dependant) SSADH from *E. coli* [1] which shares 54% identity with the human SSADH. Comparison of the two SSADH structures suggests that *E. coli* SSADH is also redox regulated, furthermore it reveals that the bacterial SSADH is structurally suited for NADP^+^, rather than NAD^+^ (as utilised by its human counterpart).

## Results and Discussion

### Production and Characterisation of E. coli SSADH

Recombinant *E. coli* SSADH was purified as a tetrameric molecule (determined by analytical size-exclusion chromatography; data not shown) which is in agreement with the previous description in the literature [6]. The conversion of SSA to SA by purified SSADH was confirmed by ^1^H NMR, as shown in Figure S1. At pH 8.0 and under the condition as described in the materials and methods, the *K_m_* of the purified enzyme is 16.94±2.2 µM and *V_max_* is 40.92±1.3 µM. The enzyme activity measured in the presence of NADP^+^ is approximately 20-fold higher than that measured in the presence of NAD^+^ (data not shown) as described previously [27].

### The X-Ray Crystal Structure of SSADH

**Figure 2:**
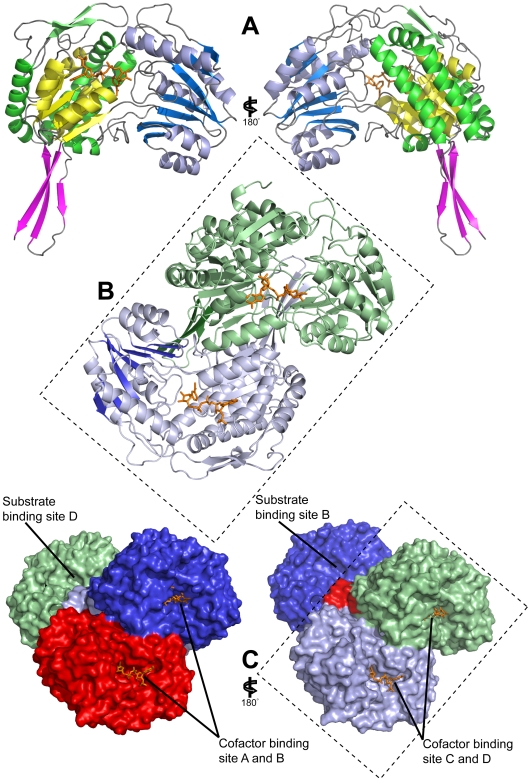
Crystal structure of *E. coli* SSADH. a) Two cartoon representations of *E. coli* SSADH monomer (rotated by 180°) with NADP^+^ bound (orange) comprises of the catalytic domain (blue and light blue) with catalytic loop (red), the cofactor binding domain (green and yellow, where yellow illustrates the Rossmann fold) and the oligomerisation domain (magenta); b) A cartoon representation of the *E. coli* SSADH dimer with NADP^+^ bound (orange), it can be seen that the 3-stranded oligomerisation domain β- sheet (dark green) of the green monomer is extending the 7-stranded catalytic domain β- sheet (dark blue) of the blue monomer to form a 10-stranded β- sheet. c) Two surface representation models of the SSADH tetramer (rotated by 180°) showing the dimer of dimer formation between the blue and red monomer and the green and light blue monomers. NADP^+^ (orange) can be seen on the same face of the dimer, the substrate binding pocket has also been labelled.

The structure of *E. coli* SSADH reveals four monomers (A–D, 481 amino acid per monomer) in the asymmetric unit (Figure 2), forming, like other members of the aldehyde dehydrogenase (ALDH) family, a biologically relevant homotetramer [28], [29], [30], [31], [32] with the 4 monomers related by a non-crystallographic 222 symmetry. The four monomers can be superposed with root-mean-square deviation (r.m.s.d: over all Cα's) of 0.193 to 0.377 Å. Monomers AB and CD form obligate dimers, which then assemble into a functional tetramer [32]. For the initial description of the structure, we refer primarily to monomer A.

The SSADH monomer (Figure 2A) adopts a typical NAD(P)^+^ dehydrogenase fold with four β sheets (A–D) and 13 α helices (1–13). The secondary structure assignments used in this report are as shown in Figure S2. The L-shaped molecule consists of three domains: The catalytic domain (residues 256–439), the cofactor binding domain (1–124, 146–254 and 446–471) and the oligomerisation domain (residues 125–147, 472–481). In each monomer, the cofactor NADP^+^ is bound to the active site giving rise to a binary complex, whereas the structure of the N-terminal His-tag was not resolved.

The catalytic domain consists of a central 7 stranded β-sheet (the D-sheet) flanked by 2 helices on one side and 3 helices on the other. The catalytic loop (residues 282–290) is located adjacent to the cofactor binding site. The cofactor binding domain interacts with NADP^+^ via two tandem Rossmann folds in a (βα)_4_β formation. This is a variation of the classic Rossmann fold [33], where the last β strand of first (βα)_2_β motif (residues 148–208) forms the first β strand of the second (βα)_2_β motif (residues 205–254). The oligomerisation domain comprises an elongated 3-stranded β-sheet (the B-sheet), which interacts with two other monomers in the final tetrameric assembly.

The obligate dimer (A+B, C+D) is formed by domain swapping; such that strand s3B of the oligomerisation domain in monomer A forms β-sheet hydrogen bonding with strand s7D of the catalytic domain in monomer B to make a 10 stranded β-sheet (Figure 2B). The screw axis of the non-crystallographic 2-fold symmetry is centred on the C β-sheet of the Rossmann fold. A total of 34 H-bonds and 13 salt bridges are made between the monomers with ∼2470 Å^2^ buried in the dimer interface (Table S1).

The tetramer can be described as a back-to-back dimer of dimer AB and CD via a 90° rotation (Figure 2C) with ∼1630 Å^2^ buried in the interface and 10 H-bonds (Table S1). Strand s1B of the oligomerisation domain of monomer A and that of monomer C sit side-by-side and the two β-sheets form a V-shape at the interface. Monomer B forms similar interactions with monomer D. Contacts form between all monomers with respect to Monomer A are listed in Table S1.

### Structural Comparison of Human and E. coli SSADH

**Figure 3:**
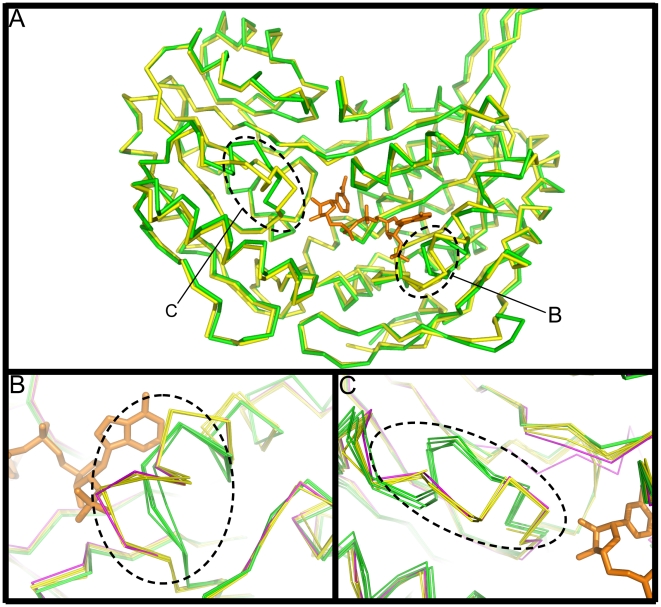
A single molecule superposition of *E. coli* SSADH and human SSADH. A) Cα trace of monomer A of *E. coli* SSADH (green) superposed with the human SSADH molecule (PDB ID: 2w8r [26]: yellow: r.m.s.d. = 0.712 over 473 residues), with the NADP^+^ moiety (orange) from *E. coli* SSADH. Two structurally variable regions have been highlighted with dashed lines and labelled B–C. Figures B–C show Cα traces of all four *E. coli* SSADH monomers A–D (green) and 5 human SSADH monomers (open loop, PDB ID: 2w8o, 2w8p, 2w8q, 2w8r yellow; closed loop, PDB ID: 2w8n magenta)[26] superposed onto each other, only one NADP^+^ molecule (orange) from monomer A of *E. coli* SSADH is shown. B) Shows the region surrounding the 3 amino acid insertion (_261_RKN_263_) in human SSADH, which clashes with the 2'phosphate of NADP^+^. C) The loop motif connecting s2D and s3D in the catalytic domain, residues A_379_–G_388_ in *E. coli* SSADH and M_432_–G_441_ in human SSADH (r.m.s.d. of 3.4 Å over 10 residues). The *E. coli* SSADH loop is conserved throughout the ALDH family and is stabilised by 7 hydrogen bonds. The novel loop in human SSADH is stabilised by only 3 hydrogen bonds, furthermore this same loop in the reduced wild type human SSADH (PDB ID: 2w8o)[26] is highly flexible and could not be determined using X-ray crystallography.

The structure of human SSADH in both the active (open, reduced; PDB ID: 2w8o) and inactive (closed, oxidised; PDB ID: 2w8n) state has recently been determined [26]. *E. coli* SSADH was purified and crystallised in the presence of the reducing agent β-mercaptoethanol, and accordingly, the structure we report most closely resembles the active form of human SSADH (2w8o) and superposes with a root-mean-square deviation of 0.79 Å over 472 Cα (2w8o and Monomer A, Figure 3A and Figure S2). The structure of the catalytic loop in *E. coli* and human SSADH is essentially identical, furthermore, the two cysteine residues involved in the redox switch in human SSADH are conserved in *E. coli* (Figure S2). These data suggest that *E. coli* SSADH may also be regulated via the redox status of the surrounding milieu. Significantly, our results show that *E. coli gabD* gene product is inactive in the presence of H_2_O_2_ and can be reactivated upon addition of DTT (Figure S3). Interestingly, the other *E. coli* SSADH gene, *sad*, does not contain the dual conserved cysteine residues in the catalytic loop and therefore it may not be regulated via the same redox mechanism.

Comparison of human and *E. coli* SSADH reveals two major regions of structural variation: the first involves the cofactor binding site (discussed below; Figure 3B). The second is the loop K380-F387 of *E. coli* (L433–F440 in human SSADH; r.m.s.d. 3.4 Å over 10 residues: Figure 3C) in the catalytic domain. The structure observed in *E. coli* SSADH (K380–F387) resembles the canonical ALDH fold [28], [29], [30], [31], [32] and permits the conserved glutamate (E385) to bind to the nicotinamide ribose moiety of NADP+. Neither the nicotinamide nor the ribose moieties were visible in electron density in the human SSADH structures: and it is likely that this mobility may impact on the L433–F440 loop.

### The Substrate and Cofactor Binding Sites in E. coli SSADH

**Figure 4:**
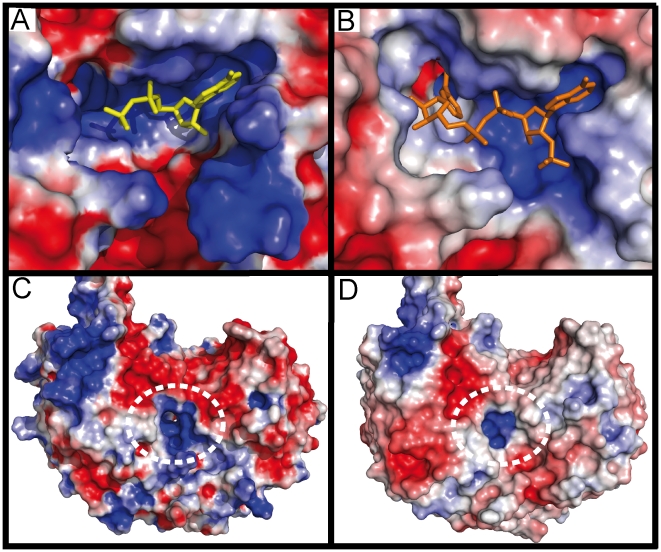
Comparisons between human (PDB ID: 2w8r)[26] and *E.coli* (monomer A) SSADH cofactor binding and SSA binding pockets, visualised using electrostatic surface representations (red represents negatively charged surfaces and blue represents positively charged surfaces). A–B) both human (A: NAD^+^ in yellow) and *E. coli* (B: NADP^+^ in orange) SSADH have a two pocket NAD(P)^+^ binding site per molecule, the first (mostly blue), positively charged and close to the surface, accommodates the adenosine moiety (and the 2'phosphate in *E. coli* SSADH). The second binding pocket, deep in the active site, houses the nicotinamide ribose moiety (absent in human SSADH). The smaller human SSADH cofactor binding pocket has a large positive protrusion, which closes the bottom of the pocket, while the larger *E. coli* SSADH cofactor binding pocket can clearly accommodate the 2'phosphate of the NADP^+^. C–D) shows the positively charged SSA binding pocket of both human (C) and *E. coli* (D) SSADH highlighted by a white dashed line. The human SSA binding pocket is larger than the *E. coli* SSA binding pocket.

In each SSADH monomer, the catalytic residues are located at the centre of the molecule with two funnel-like openings on the surface of either side of the molecule. The larger opening functions to allow entry of the cofactor NADP^+^ (Figure 4A, B). On the opposite side of the monomer, the smaller opening is located, and this cavity is utilized for substrate entry and product exit (Figure 4C, D, 2C) as for other ALDHs.

**Figure 5:**
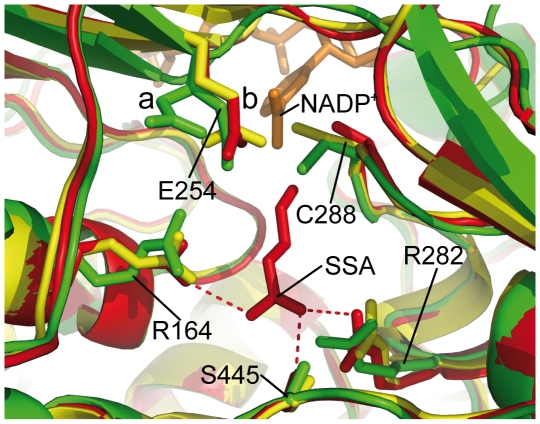
SSADH substrate binding and the active site. A cartoon representation of the *E. coli* SSADH (monomer A: green) substrate (SSA) binding pocket superposed onto human SSADH C340A mutant with SSA bound (PDB ID: 2w8q [26]: red) and human SSADH containing the catalytic cysteine (PDB ID: 2w8o [26]: yellow). The key SSA binding residues from 2w8q have their interactions with SSA shown as a red dashed line. Superposition of catalytic residues are shown: the catalytic cysteine and the general base as sticks (labeled according the *E. coli* SSADH) and NADP^+^ (orange) from *E. coli* SSADH. It can be seen that the equivalent SSA binding residues from 2w8o and *E. coli* SSADH (R164, R283 and S445) are in a very similar location and orientation to those of 2w8q. The catalytic cysteine of 2w8o is oriented toward the NADP^+^ moiety while in *E. coli* C288 is oriented toward the substrate (SSA). Also two conformations of the general base (E254) can be seen in *E. coli* SSADH, with (a) being in the hydride conformation and (b) the hydrolysis conformation.

*E. coli* SSADH was crystallised in the presence of both NADP^+^ and SSA. We observe no electron density for the substrate. However, superposition between human and *E. coli* SSADH does permit us to identify the substrate binding pocket (Figure 5).

The conserved catalytic residues (C340 and E306) and the active site residues (R213, R334 an S498) of human SSADH structures (PDB ID: 2w8o and 2w8q)[26] superpose well with that of *E. coli* SSADH (C288 and E254; R164, R282 and S445 respectively, Figure 5, Figure S2) [26].

### Catalytic Mechanism of SSADH

The catalytic mechanism for ALDH enzymes is well characterised. The first step of the reaction is nucleophilic attack by the catalytic C288 residue on SSA to give the hemithioacetal intermediate. Hydride transfer from this intermediate to NAD(P)^+^ results in formation of the thioacyl enzyme intermediate and NAD(P)H. Lastly, the conserved E254 residue acts as a general base to deprotonate a water molecule prior to its attack on the thioacyl enzyme intermediate resulting in formation of SA and regeneration of the C288 residue. The general base, E254, in all monomers can be modelled in two alternative conformations according to the electron density. It is suggested that the two conformations of E254 is likely to be associated with different stages of the catalytic process, with one conformation “a” (Figure 5) being for hydride transfer and conformation “b” for hydrolysis.

**Figure 6:**
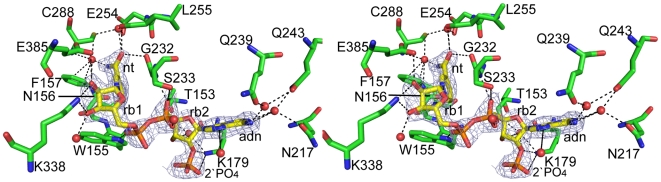
NADP^+^ binding of *E. coli* SSADH. Stereo view of the active site showing the NADP^+^ moiety (yellow), SSADH residues (green) involved in binding NADP^+^, water molecules can be seen as red spheres and all bonds are depicted with a black dashed line. The 2*F_0_–F_c_* omit electron density of the NADP^+^ moiety contoured at 1σ is also shown (light blue mesh). Interactions of monomer A and NADP^+^ can be seen, specifically both AN1 and AN6 of the adenine moiety (labelled adn) interacts with Q239(O^ε1^), Q243(O^ε1^) and N217(O^δ1^) via water molecules. Adjacent to the adenine moiety, both AO2 and AO3 of the ribose (labelled rb2) hydrogen bonds with T153(O) and K179(N^Z^, O) via a single water molecule. 3AOP of the 2'-phosphate interacts with K179(N^Z^). AO2 of the pyrophosphate interacts with S233(N, O^G^) and the NO1 hydrogen bonds directly with W155(N^ε1^). Both NO2 and NO3 of the adjacent ribose moiety (labelled rb1) hydrogen bonds with K338(N^Z^), while NO2 also interacts with E385(O^ε1^). NN7 of the nicotinamide (labelled nt) moiety interacts with N156(N^δ2^) and the catalytic C288(N) via the single water molecule. While NO7 interacts directly with G232(O) and L255(O), as well as with L255(N) and E254(O^ε1^) via the same water molecule. Up to 13 SSADH residues make 24 van der Waals or hydrogen bonds interactions with NADP^+^ per monomer, 16 of which are mediated by water (Table S2). Notably, all of the residues involved directly NADP^+^ binding in *E. coli* SSADH [48] (Table S2) are also conserved in human SSADH.

In the *E. coli* SSADH structure, electron density for the cofactor in the binding sites is clearly evident (Figure 6). Surface representation of the cofactor binding site illustrates where the cofactor is positioned; also shown is the human structure (PDB ID: 2w8r)[26] in which the cofactor NAD^+^ is soaked into the binding site of the C340A mutant (Figure 4A, B). The cofactor binding site comprises two pockets; one of which is close to the surface of the SSADH molecule and accommodates the adenosine (adenine and the first ribose) and the 2'phosphate. The second pocket is located centrally in the active site and accommodates the second ribose and the nicotinamide.

A key difference between these two enzymes is that human SSADH utilizes NAD^+^ as a cofactor, whereas *E. coli* SSADH utilizes NADP^+^. Our structural data reveals the basis for this preference - in *E. coli* SSADH a three-residue deletion (of the human sequence _261_RKN_263_, Figure S2) in the loop connecting s5C and h6 permits accommodation of the extra phosphate group of NADP^+^ (2'phosphate: Figure 3B, 4A and B). Interestingly, although *E. coli* SSADH can utilise NAD^+^ as a cofactor the activity in the presence of this molecule is only 1/20 of that of NADP^+^ (data not shown). In human SSADH, _261_RKN_263_ (Figure 3B and 4A) occupies the space for the 2'phosphate of NADP^+^; consequently, only NAD^+^ but not NADP^+^ can be utilised as a cofactor for this enzyme.

Importantly our structural data permits unambiguous placement of the entire NADP^+^ moiety including that of the nicotinamide ring in each active site (Figure 6, Table S2). This is in contrast to other published ALDH structures (including the human SSADH structures) where the nicotinamide ring is often partially disordered, indicating flexibility. In our structure, the NO2 and NO3 of the nicotinamide ribose interacts with E385 and K338 respectively; while NO7 of the nicotinamide ring binds to the backbone of L255 and G232 of the catalytic domain (Figure 6, Table S2). Such interactions are rarely observed, in particular, this region is disordered (suggesting flexibility) in the human SSADH structure (PDB ID: 2w8r)[26].

**Figure 7:**
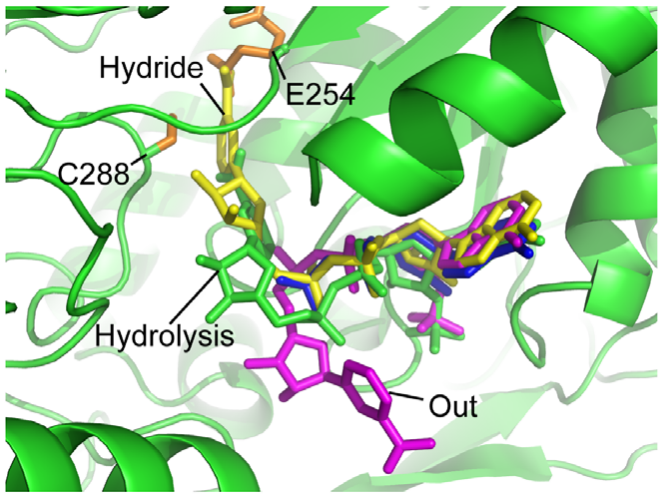
Different binding states of NAD(P)^+^ in the ALDH family. Four different conformations of NAD(P)^+^ are shown as sticks, hydride conformation (PDB ID: 1bpw[31]: yellow), hydrolysis conformation (*E. coli* SSADH: green), out conformation (PDB ID: 2ilu [29]: magenta) and flexible, where the nicotinamide ribose moiety is unable to be resolved using X-ray crystallography (PDB ID: 2w8r [26]: blue). The general base (E254) and the catalytic cysteine (C288: both orange), which are conserved in human and *E. coli* SSADH and the whole ALDH family, have been labelled to define the active site.

Amongst the well defined cofactor crystal structures, three different conformations of NAD(P)^+^ found in the ALDH superfamily have been described [29]–the hydride transfer (PDB ID: 1bpw [31]), the hydrolysis (see below) and the “out” conformation (PDB ID: 2ilu [29]; Figure 7). Superposition with ALDH structures in the hydrolysis conformation (PDB ID: 1bxs and 1O01 [34], [35]) reveals that the cofactor in the *E. coli* SSADH structure most closely resembles this conformation, where the nicotinamide ring is retracted from the active site such that the general base E251 is now situated in an ideal distance (3.69 Å to the substrate SSA and 3.15 Å to the catalytic cysteine C288) to catalyse the deacylation process.

### Analysis of E. coli SSADH Structure with Respect to Human Disease-Linked Mutations

**Figure 8:**
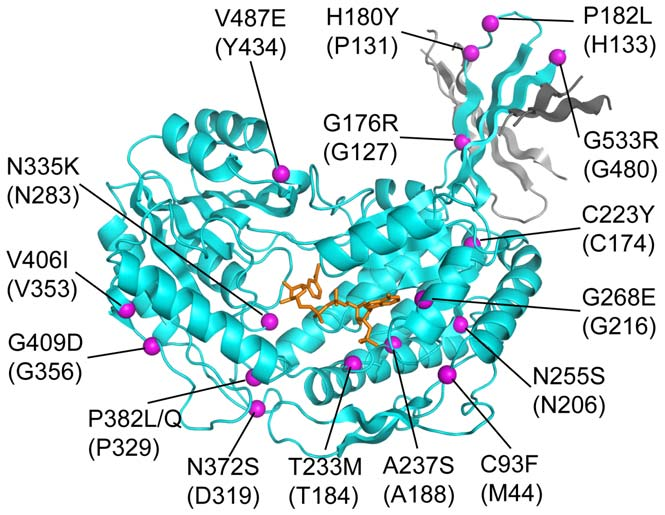
Human point mutations mapped onto the *E. coli* SSADH structure. A cartoon representation of *E. coli* SSADH monomer A (cyan, NADP^+^ orange) showing the 17 point mutations (magenta spheres) that map to the mature human protein. A small region of monomer B catalytic domain (dark grey) and monomer C oligomerisation domain (light grey) have been included to illustrate the proximity of point mutations with regard to dimer and tetramer interfaces. The mutations occur in all three domains, 4 in the oligomerisation domain (G176R, H180Y, P182L and G533R), 6 in the cofactor binding domain (C93F, C223Y, T233M, A237S, N255S and G268E) and 7 in the catalytic domain (N335K, N372S, P382L/Q, V406I, G409D and V487E).

To date, more than 40 mutations found in patients with SSADH deficiency (Table 1 & 2) have been documented in the literature [9], [36], [37], [38], [39], [40], [41] (in this work, all numbering of point mutations use human SSADH numbering with *E. coli* SSADH numbering in parentheses). The majority (25 mutations) give rise to truncations, deletions, insertions as well as splice site mutants (Table 1). However, eighteen point mutations (all missense) are found in the coding sequence, one of which (G36R) is located at the mitochondrial targeting sequence (Table 2). The remaining 17 variants are mapped onto the *E. coli* SSADH structure (Figure 8, Table 2). Of these, six mutations (G36R, H180Y, P182L, A237S, N372S and V406I) are most likely non-pathogenic [42]. Of the mutations associated with disease, five mutations map to four positions in the catalytic domain (N335K, P382L/Q, G409D, V487E); four are found in the cofactor binding domain (C223Y, T233M, N255S and G268E); and two are mapped to the oligomerisation domain (G176R and G533R). Mutations that lead to a dramatic decrease of enzyme activity (2% or less of the wildtype) are found to be strictly conserved between human/*E.coli* SSADH (G176R/G127, G268E/G216, N335K/N283, P382L/P329, G409D/G356, G533R/G480). All these residues superpose well between the human and *E. coli* structures (Figure S4).

**Table 1:** Mutations causing nonsense mutations of SSADH from SSADH deficient patients.

Mutation type	Mutation in protein	Resulting protein
Nonsense mutations leading to truncation	Q79Stop	11 residues of N-term.
	Y128 Stop	60 residues of N-term.
	K192 Stop	124 residues of N-term.
	W204 Stop	136 residues of N-term.
	R261 Stop	206 residues of N-term.
	R412 Stop	357 residues of N-term.
	R514 Stop	459 residues, missing one strand in the oligomerisation domain only.
Deletions	S35fs X49 Stop	Missing all residues of the mature protein.
	S55fs X79 Stop	Missing all residues of the mature protein.
	H154fs X10 Stop	86 residues of N-term.
	S208fs X2 Stop	140 residues of N-term.
	V392fs X10 Stop	337 residues, Missing half of the catalytic and one strand of the oligomerisation domains and helix 13 of the cofactor binding domain.
	A188fs X8 Stop	120 residues of N-term.
	G488fs X5 Stop	Missing last strand of the catalytic and oligomerisation domains and helix 13 of the cofactor binding domain.
Splice site mutations	E119-K290 deletion	Missing entire Rossmann fold and catalytic loop.
	K148fs X16 Stop	80 residues N-term.
	Exon 5 deleted, L243-K290 deleted	Missing last strand of the Rossmann fold and the catalytic loop.
	Exon 5 deleted, L243-K290 deleted	Missing last strand of the Rossmann fold and the catalytic loop.
	F449fs X53 Stop	394 residues, Missing 2 strands and 2 helices of the catalytic and one strand of the oligomerisation domains and helix 13 of the cofactor binding domain.
	Exon 7 deleted, 292-344 deleted	Missing 2 helices and one strand from the catalytic domain.
	Exon 9 deleted, 401fs X52 Stop	Missing 2 strands and 2 helices of the catalytic and one strand of the oligomerisation domains and helix 13 of the cofactor binding domain.
Insertions	C93-R99 duplication	Duplication in loop between s2A and the start of helix h1.
	A12 fs X123 Stop	Missing entire mature protein
	A153fs X12 Stop	85 residues N-term.
	P442fs X18 Stop	Missing 3 strands and 2 helices of the catalytic and one strand of the oligomerisation domains and helix 13 of the cofactor binding domain.

fs = frameshift; X = number of missense residues after the frameshift; Stop = stop codon, termination of the protein.

Resulting SSADH protein size has been calculated excluding the 47 amino acid N-terminal mitochondrial signal peptide, the mature protein size is 488 residues. All mutations previously published [9], [36], [37], [38], [40], [41], [42], [60], [61].

**Table 2:** Analysis of point mutations found in human SSADH enzymes as a result of missense mutations.

Mutated Residue	% Activity of WT (previously published)	Change in residue properties	Residue in *E. coli* SSADH	Molecule location in *E. coli* SSADH	Residue function in *E. coli* SSADH
G36R^A^	87%	Small polar to large basic polar	N/A	N/A	N/A
C93F^B^	3.0%	Small non-polar to large non-polar	M44	Loop between s2A & h1	Possible interaction with Rossmann fold
G176R^B^	<1.0%	Small polar to large basic polar	**G127**	Strand s1B	Intermolecular tetramer contact
H180Y^AC^	83%	Both large intermediate polarity	P131	Loop between s1B & s2B	Intermolecular contact region
P182L^AC^	48%	Small non-polar to large non-polar	H133	Loop between s1B & s2B	Intermolecular dimer contact
C223Y^B^	5.0%	Small non-polar to large intermediate polarity	**C174**	Loop between h4 & s4C	Rossmann fold
T233M^B^	4.0%	Small non-polar to large non-polar	**T184**	Loop between s4C & h5	Rossmann fold
A237S^A^	65%	Small non-polar to small polar	**A188**	Helix h5	Rossmann fold
N255S^B^	17%	Both small polar	**N206**	Strand s5C	Rossmann fold
G268E^B^	<1.0%	Small polar to large acidic polar	**G216**	Helix h6	Rossmann fold
N335K^B^	1.0%	Small polar to large basic polar	**N283**	Loop between h8 & s4D	Catalytic loop
N372S^C^	n.d.	Both small polar	D319	Loop between h9 & h10	Catalytic domain
P382L^B^	2.0%	Small non-polar to large non-polar	**P329(a)**	Loop between h9 & h10	Catalytic domain
P382Q^B^	n.d.	Small non-polar to larger polar	**P329(b)**	Loop between h9 & h10	Catalytic domain
V406I^C^	n.d.	Both non-polar	**V353**	Strand s1D	Catalytic domain
G409D^B^	<1.0%	Small polar to small acidic polar	**G356**	Loop between s1D & s2D	Catalytic domain
V487E^B^	n.d.	Large non-polar to large acidic polar	Y434	Strand s7D	Intermolecular contact region
G533R^B^	<1.0%	Small polar to large basic polar	**G480**	Strand s3B	Intermolecular contact

**Bold** denotes conserved residue between human SSADH and *E. coli* SSADH.

n.d. = not determined.

A Denotes non-pathogenic point mutations found in patients with SSADH deficiency.

B Denotes pathogenic point mutations found in patients with SSADH deficiency.

C Allelic polymorphisms found in the general population

All mutations and reaction rates previously published [9], [36], [42], [60].

### Conclusions

SSADH plays an essential role in living organisms including the central nervous system, both in development and in cognitive function [43]. However, relatively little is known about the chemistry of the active site of SSADH. In the present study, we begin to address this problem by determining the 2.3 Å X-ray crystal structure of SSADH from *E. coli.*

One key difference between human and *E. coli* SSADH is that the human enzyme utilises NAD^+^ as a cofactor, whereas the bacterial counterpart uses NADP^+^. Interestingly, analysis of sequence alignments reveals a single sequence insertion event of three amino acids (R206, K207, N208, Figure S2) in human SSADH. This insertion maps to the loop between Strand s5C and α6 in the Rossmann fold which in *E. Coli* SSADH forms the pocket that binds the 2'phosphate group of NADP^+^. Given that NAD^+^ does not contain the 2'phosphate group, we postulate that the insertion of the two positively charged residues may restrict the adenosine-binding pocket of human SSADH to bind NAD^+^ rather than NADP^+^. Related to this observation, we also note that a splice variant of human SSADH has been characterised that involves a 12 amino acid substitution and a shortening of the h5 helix. While the expression levels of this variant has not been characterised we speculate that it is possible that this substitution would open up the adenosine- binding pocket and may permit binding of the alternative cofactor NADP^+^.

Our structural data also permit us to analyse human SSADH mutations that cause disease, our analysis reveals that human point mutations associated with SSADH deficiency mutations cluster in three key areas. One group of mutations affect the cofactor binding domain, a second group directly impact on the catalytic domain and, finally, several mutations involve residues that appear to be important for formation of the dimer and/or the tetramer. The latter mutations are of particular interest, since they demonstrate how important homo-tetramerisation is for biological activity. It is important to note, however, that each monomer contains a complete catalytic unit, i.e. that no part of the catalytic machinery is contributed *in trans* from another monomer. The precise contribution of SSADH tetramer formation to its biological function remains to be understood. However, it is worth noting that many members of the ALDH family are allosterically regulated (see for example [44]).

To conclude, our work provides additional structural insight into an important enzyme that in humans regulates metabolism of the neurotransmitter glutamate and GABA. Perturbation of GABA levels have been linked to many different neurological diseases, including depression and movement disorders [45]. Therefore there is much clinical interest in enzymes that impact on levels of GABA and related molecules. The role of SSADH appears to primarily metabolise a toxic by-product of glutamate and GABA metabolism–SSA and, accordingly inhibiting its activity would be anticipated to have deleterious effects. However, the tetrameric nature of SSADH as well as analysis of related molecules suggest that SSADH may be allosterically regulated. Such a feature, if supported through experimental data, may potentially be useful to improve SSADH function and SSA degradation in cases of partial SSADH deficiency.

## Materials and Methods

### Gene Cloning, Expression and Purification

The cDNA encoding SSADH was isolated from *E. coli* MC1061 genomic DNA using PCR with the following primers 5′ccagaattcaatgaaacttaacgacagtaac and 5′cccagatctaagcttaaagaccgatgcacatatatttg and then cloned into pCR® -Blunt (Invitrogen). The DNA sequence of the PCR product was shown to have a single amino acid change to that of the *gabD* gene sequence in the database, which is thought to represent a naturally occurring variant in *E. coli*, the SSADH cDNA was excised from the recombinant pCR® -Blunt vector using the restriction enzymes *EcoR*I and *Hind*III, then ligated into pRSETc/His_TEV plasmid as previously described [46]. The recombinant plasmid was transformed into *E. coli* BL21(DE3) pLysS cells and the transformant was stored at −80°C. For expression of SSADH recombinant protein, transformed *E. coli* BL21(DE3) cells were propagated in 2YT growth medium in the presence of 100 µg/mL ampicillin and 36 µg/ml chloramphenicol at 37°C to A_600_ = 0.6 followed by induction at 16°C with 0.5 mM IPTG for 18 hours.

Harvested cells were treated with lysozyme (1 mg/ml) at 4°C for 30 min and lysed in lysis buffer containing 300 mM NaCl, 10 mM imidazole, 5 mM β-mercaptoethanol, 0.01% triton X-100, 50 mM Tris (pH 8.0) by sonication on ice. Clarified cell lysate was loaded onto a Nickel Chelating Sepharose Fast Flow column (GE healthcare), the His-tagged SSADH protein was eluted in 150 mM NaCl, 250 mM imidazole, 5 mM β-mercaptoethanol, 50 mM Tris (pH 8.0). Purified His-tagged SSADH were further purified using a S200 16/60 size exclusion column (GE Healthcare) pre equilibrated with 100 mM NaCl, 10 mM β-mercaptoethanol, 5% glycerol, 30 mM Tris (pH 7.5) for all experiments described in this paper.

### Enzyme Kinetics and NMR Studies

Enzyme kinetics were carried out using purified SSADH (2 µg/ml) in a Na phosphate buffer (100 mM pH 8.0), containing 1.1 mM NADP^+^ and SSA 0–400 mM at 30°C. The rate at which NADP^+^ was converted to NADPH was monitored fluorometrically (excitation: 355 nm and emission: 460 nm). SSADH oxidative inhibition (H_2_O_2_) assays were carried out by incubating various concentrations of H_2_O_2_ with SSADH (1 µg/ml) in a Na phosphate buffer (100 mM pH 8.4), containing 0.75 mM EDTA for 1 hour at room temperature, the reactions were terminated by adding 5.0 mM methionine. To reverse the effect of the H_2_O_2_, 10 mM DTT was added to H_2_O_2_ treated SSADH and incubated for a further 10 minutes. After the addition of 2.0 mM NADP^+^ and 0.15 mM SSA, the activity of H_2_O_2_ treated SSADH was measured spectrophotomically at 340 nm at 30°C.

^1^H NMR spectroscopic analysis was carried out with a Bruker DPX 400 MHz spectrometer using purified SSADH in the presence of 125 µM SSA for 240 min as above.

### Crystallisation and Data Collection

Pure SSADH was concentrated to 6 mg/mL and buffer exchanged into 20 mM NaCl, 10 mM β-mercaptoethanol, 5% glycerol, 1.0 mM NADP, 1.0 mM succinic semialdehyde, 30 mM Tris (pH 7.5) using a 10 KDa molecular weight cut off concentrator (Millipore).

Crystals of SSADH were grown using hanging drop vapour diffusion by mixing 2.0 µL of protein (6 mg/mL) with 1.0 µL of reservoir solution containing 0.2 M ammonium tartrate, 26–31% polyethylene glycol 3350, 10 mM β-mercaptoethanol and 0.1 M Tris (pH 7.2–7.5). Crystals were dehydrated for 48 hours in 0.2 M ammonium tartrate, 32% polyethylene glycol 3350, 10 mM β-mercaptoethanol and 0.1 M Tris (pH 7.2–7.5) after three days. Crystals were soaked in reservoir solution supplemented with 5% 2-methyl-2,4-pentanediol for 5 minutes then soaked for a further 5 minutes in reservoir solution supplemented with 10% 2-methyl-2,4-pentanediol for cryoprotection before freezing in liquid nitrogen for data collection. Data were collected at the Australian Synchrotron High-throughput protein crystallography (PX1) beam.

### Structure Determination and Refinement

SSADH crystals diffracted to 2.3 Å resolution and belong to the space group *P*42_1_2 with unit cell dimensions of *a* = 151.88 Å, *b* = 151.88 Å, *c* = 165.77 Å, α = β = γ = 90.0°. These data are consistent with four molecules in the asymmetric unit. These data were merged and scaled using MOSFLM [47] and SCALA [48]. Subsequent crystallographic and structural analysis was done using the CCP4i interface to the CCP4i suite unless stated otherwise. Five percent of the data was flagged for R*_free_* with neither a sigma nor a low-resolution cut-off applied to the data. Summaries of the statistics are provided in Table 3. The completeness of the data is relatively low (87%) due to overlaps caused by close spots, a result of the beam being centred on the long cell edge of the unit cell.

**Table 3:** Data collection and refinement statistics*.

Data collection statistics	
Space group	*P*42_1_2
Cell dimensions	
a, b, c (Å)	151.9, 151.9, 165.8
α, β, γ, (Å)	90, 90, 90
Resolution limit	2.30–37.56 (2.30–2.42)
R_pim_	4.4% (23.4%)
R_merge_	18.8% (80.5%)
I/(σI)	16.7 (3.2)
Completeness	86.6% (77.5%)
Multiplicity	17.4 (11.4)
Refinement statistics	
Resolution (Å)	2.3
Total n°. of obs.	1300495
Total n°. unique	74567
R_work_/R_free_	17.0% 21.4%
No. Atoms	
Protein	14709
Ligand	192
Water	527
R.M.S deviations	
Bond lengths (Å)	0.010
Bond angles (°)	1.3
Molprobity score	8.45 (96^th^ percentile)

*Values in parenthesis are for the highest resolution shell.

The structure of SSADH was solved using molecular replacement and the program PHASER [49]. A five-model ensemble was constructed in PHASER using the structures that possess closest sequence identity to SSADH (identified using the FFAS server [50]: 5-carboxymethyl-2-hydroxymuconate semialdehyde (PDB ID: 2d4e; unpublished), aldehyde dehydrogenase A (PDB ID: 2hg2)[29], aldehyde dehydrogenase (PDB ID: 1bxs)[34], formyltetrahydrofolate dehydrogenase (PDB ID: 2o2p)[51] and putative betaine aldehyde dehydrogenase (PDB ID: 1wnb)[30].

A ‘mixed’ model consisting of conserved side chains with all other non-alanine/glycine residues truncated at Cγ atom was then created using the SCRWL server [52]. The five-model ensemble was used as a search model in PHASER and a model based on 2o2p with an initial solution with a Z score of 44.8 that packed well within the unit cell was identified. Together with the unbiased features in the initial electron density, these data suggested a correct molecular replacement solution.

Refinement and model building preceded using one molecule (chain A) in the asymmetric unit, with the other chains built using non-crystallographic symmetry operators. Maximum likelihood refinement using REFMAC[53] was carried out using bulk solvent correction (Babinet model). Tight NCS restraints were imposed on all residues in the four molecules in the asymmetric unit, NCS restraints were relaxed in flexible regions as suggested by monitoring of the *R*_free_. Model building and structural validation was performed using COOT [54]. Water molecules were added to the model using ARP/warp [55] when the *R*_free_ reached 28%. The presence of each water molecule was manually validated. The NADP^+^ moiety was modelled into the density using PHENIX ligandfit [56], [57]. The stereochemical qualities of the final model was checked by MolProbity [58] (96^th^ percentile; Table 3).

### Accession Numbers and Datasets

Coordinates and structure factors have been deposited in the Protein Data Bank with accession number 3jz4.

Diffraction image datasets and refinement logs are available online on TARDIS (http://hdl.handle.net/102.100.100/42) [59].

## Supporting Information

Figure S1 The conversion of SSA to SA using 1H NMR spectroscopy. NMR spectra of (a) substrate alone in phosphate buffer which contains succinic semialdehyde (X), 4,4-dihydroxybutanoic acid (Y) and succinic acid (Z). (b) Succinic acid alone with phosphate buffer, showing only the singlet peak of succinic acid (Z). For SSADH enzyme assay, incubation of succinic semialdehyde and SSADH in the presence of NADP+ at 0 min (c) and 240 min (d) showing all substrates (X, Y, Z) has been converted to succinic acid (Z), NADP+ is marked with W.
(0.21 MB TIF)

Figure S2 Alignment of E. coli SSADH with human SSADH. Conserved residues have been highlighted according to the following, polar (green), non-polar (yellow), acidic (red) and basic (blue). The secondary structure (E. coli SSADH above the sequence and human below the sequence) has been marked with either an arrow designating a Î^2^-sheet or a cylinder representing an Î±-helix. The secondary structure elements are coloured according the Figure 2a. Structurally important regions have also been marked and labelled, catalytic loop (red line) and the GXXXXG motif (box) from the Rossmann fold. The human SSADH mitochondrial targeting sequence is labelled and shown as a blue line.
(1.46 MB TIF)

Figure S3 SSADH activity in an oxidised and reduced environment. The first column shows the untreated activity of SSADH. In the second column the E. coli SSADH enzyme was oxidised by incubating it with 200 Î¼M H2O, which has 11–13% of untreated SSADH activity. In the third column the addition of 10 mM DTT to previously oxidised enzyme, which rescued the inhibition to 80% that of the normal activity.
(0.16 MB TIF)

Figure S4 A–F. Human point mutations causing a dramatic loss of acticvity (≤2% residual SSADH) mapped onto the E. coli SSADH structure. A cartoon representation of E. coli SSADH monomer A (cyan, NADP+ orange) showing the 17 point mutations (magenta spheres) that map to the mature human protein. A small region of monomer B catalytic domain (dark grey) and monomer C oligomerisation domain (light grey) have been included to illustrate the proximity of point mutations with regard to dimer and tetramer interfaces. Labelled, dashed red boxes highlight the area of E. coli SSADH that have been enlarged for analysis. A–F) Show the equivalent E. coli SSADH residues (magenta sticks) to the human point mutants and their interactions (black dashed lines) within their immediate surrounds with other residues (orange sticks). A–B) Mutations in this region would be anticipated to disrupt dimer and tetramerisation, while C) we would anticipate decreased stability in this region due to loss of stabilising interactions with the N255S mutation. D) A loss of NAD/P+ binding efficiency would be expected with the addition of a large negatively charged residue, G268E, into the positively charged adenosine-ribose binding pocket of SSADH. E) We anticipate an overall loss of structural integrity of this loop region from either of the mutations G409D (loss of +/+ backbone conformation) and P382L/Q (disruption of aromatic ring stacking interactions with F419). F) The introduction of a large charged residue (N335K) in a buried surface on the catalytic loop would be anticipated to greatly disrupt the structural integrity and catalytic ability of SSADH.
(2.71 MB TIF)

Table S1 Intermolecular contacts with respect to monomer A.
(0.18 MB DOC)

Table S2 Residues involved in significant NADP+ binding using Molprobity [58].
(0.07 MB DOC)

## References

[1] Chambliss K, Caudle DL, Hinson DD, Moomaw CR, Slaughter CA (1995). Molecular Cloning of the Mature NAD+-dependent Succinic Semialdehyde Dehydrogenase from Rat and Human.. The Journal of Biological Chemistry.

[2] Blaner WS, Churchich JE (1980). The binding of NADH to succinic semialdehyde dehydrogenase.. European journal of Biochemistry.

[3] Chambliss K, Gibson KM (1992). Succinic semialdehyde dehydrogenase from mammalian brain: subunit analysis using polyclonal antiserum.. International journal of biochemistry & cell biology.

[4] Lee B, Hong JW, Yoo BK, Lee SJ, Cho SW (1995). Bovine brain succinic semialdehyde dehydrogenase; purification, kinetics and reactivity of lysyl residues connected with catalytic activity.. Molecules and cells.

[5] Ryzlak M, Pietruszko R (1988). Human brain “high Km” aldehyde dehydrogenase: Purification, characterisation, and identification as NAD+ dependent succinic semialdehyde dehydrogenase.. Archives of biochemistry and biophysics.

[6] Donnelly M, Cooper RA (1981). Two succinic semialdehyde dehydrogenases are induced when E. coli K-12 is grown on gamma-aminobutyrate.. Journal of bacteriology.

[7] Koh Y, Joo CN, Choi SY, Kim DS (1994). Purification and properties of succinic semialdehyde dehydrogenase from Lumbricus rubellus.. Journal of molecular biology.

[8] Sanchez M, Fernandez J, Martin M, Gibello A, Garrido-Pertierra A (1989). Purification and properties of two succinic semialdehyde dehydrogenases from Klebsiella pneumoniae.. Biochim Biophys Acta.

[9] Blasi P, Boyl PP, Ledda M, Novelletto A, Gibson KM (2002). Structure of human succinic semialdehyde dehydrogenase gene: identification of promoter region and alternatively processed isoforms.. Molecular Genetics and Metabolism.

[10] Fenalti G, Law RHP, Buckle AM, Langendorf C, Tuck K (2007). GABA production by glutamic acid decarboxylase is regulated by a dynamic catalytic loop.. Nature Structure and Molecular Biology.

[11] Jakoby W, Scott EM (1958). Aldehyde Oxidation: Succinic Semialdehyde Dehydrogenase.. The Journal of Biological Chemistry.

[12] Schaller M, Schaffhauser M, Sans N, Wermuth B (1999). Cloning and expression of succinic semialdehyde reductase from human brain.. European Journal of Biochemistry.

[13] Jakobs C, Jaeken J, Gibson KM (1993). Inherited disorders of GABA metbolism.. Journal of Inherited Metabolic Disorders.

[14] Pearl P, Novotny EJ, Acosta MT, Jakobs CJ, Gibson KM (2003). Succinic semialdehyde dehydrogenase deficiency in children and adults.. Annal of neurology.

[15] Gibson K, Sweetman L, Nyhan WL, Jakobs C, Rating D (1983). Succinic semialdehyde dehydrogenase deficiency: an inborn error of gamma-aminobutyric acid metabolism.. Clinica chimica acta.

[16] Gibson K, Hoffmann GF, Hodson AK, Bottiglieri T, Jakobs C (1998). 4-Hydroxybutyric acid and the clinical phenotype of succinic semialdehyde dehydrogenase deficiency, and inborn error of GABA metabolism.. Neuropediatrics.

[17] Gibson K, Gupta M, Pearl PL, Tuchman M, Vezina LG (2003). Significant behavioural disturbances in succinic semialdehyde dehydrogenase (SSADH) deficiency (Gamma-Hydroxybutyric Aciduria).. Biological psychiatry.

[18] Knerr I, Pearl PL, Bottiglieri T, Snead OC, Jakobs C (2007). Theraputic concepts in succinate semialdehyde dehydrogenase (SSADH; ALDH5a1) deficiency (γ-hydroxybutyric aciduria). Hypothese evolved from 25 years of patient evaluation, studies in Aldh5a1-/- mice and characterization of γ-hydroxybutyric acid pharmacology.. Journal of inherited metabolic disease.

[19] Cho S, Song MS, Kim YG, Kang WD, Choi EY (1993). Kinetics and mechanism of an NADPH-dependent succinic semialdehyde reductase from bovine brain.. European journal of biochemistry.

[20] Novonty EJ, Fulbright RK, Pearl PL, Gibson KM, Rothman DL (2003). Magnetic resonance spectroscopy of neurotransmitters in human brain.. Annals of neurology.

[21] Skinner M, Cooper RA (1982). An Escherichia coli mutant defective in the NAD-dependent succinate semialdehyde dehydrogenase.. Archives of Microbiology.

[22] Bartsch K, von Johnn-Marteville A, Schulz A (1990). Molecular analysis of two genes of the *Escherichia coli gab* cluster: nucleotide sequence of the glutamate: succinic semialdehyde transaminase gene (*gabT*) and characterization of the succinic semialdehyde dehydrogenase gene (*gabD*).. Journal of Bacteriology.

[23] Metzer E, Halpern YS (1990). In vivo cloning and characterization of the gabCTDP gene cluster of Escherichia coli K-12.. Journal of Bacteriology.

[24] Niegemann E, Schulz A, Bartsch K (1993). Molecular organization of the Escherichia coli gab cluster: nucleotide sequence of the structural genes gabD and gabP and expression of the GABA permease gene.. Archives of Microbiology.

[25] Fuhrer T, Chen L, Sauer U, Vitkup D (2007). Computational Prediction and Experimental Verification of the Gene Encoding the NAD+/NADP+-Dependent Succinate Semialdehyde Dehydrogenase in *Escherichia coli*.. Journal of Bacteriology.

[26] Kim Y, Lee S, Kwon OS, Park SY, Lee SJ (2009). Redox-switch modulation of human SSADH by dynamic catalytic loop.. The EMBO Journal.

[27] Jaeger M, Rothacker B, Ilg T (2008). Saturation transfer difference NMR studies on substrates and inhibitors of succinic semialdehyde dehydrogenases.. Biochemical and Biophysical Research Communications.

[28] Cobessi D, Tete-Favier F, Marchal S, Azza S, Branlant G (1999). Apo and holo crystal structures of an NADP-dependent aldehyde dehydrogenase from *Streptococcus mutans*.. Journal of Molecular Biology.

[29] Di Costanzo L, Gomez GA, Christianson DW (2007). Crystal structure of lactaldehyde dehydrogenasse from *Escherichia coli* inferences regarding substrate and cofactor specificity.. Journal of Molecular Biology.

[30] Gruez A, Roig-Zamboni V, Grisel S, Salomoni A, Valencia C (2004). Crystal structure and kinetics identify *Escherichia coli* YdcW gene product as a medium-chain aldehyde dehydrogenase.. Journal of Molecular Biology.

[31] Johansson K, El-Ahmad M, Ramaswamy S, Hjelmqvist L, Jornvall H (1998). Structure of betaine aldehyde dehydrogenase at 2.1 Å resolution.. Protein Science.

[32] Steinmetz C, Xie P, Weiner H, Hurley TD (1997). Structure of mitochondrial aldehyde dehydrogenase: the genetic component of ethanol aversion.. Structure.

[33] Weiner H, Hurley TD (2004). NADP+ binding to dehydrogenases.. Encyclopedia of Life Sciences.

[34] Moore S, Baker HM, Blythe TJ, Kitson KE, Kitson TM (1998). Sheep liver cytosolic aldehyde dehydrogenase: the structure reveals the basis for the retinal specificity of class 1 aldehyde dehydrogenases.. Structure.

[35] Perez-Miller S, Hurley TD (2003). Coenzyme Isomerization Is Integral to Catalysis in Aldehyde Dehydrogenase.. Biochemistry.

[36] Aoshima T, Kajita M, Sekido Y, Ishiguro Y, Tsuge I (2002). Mutation analysis in a patient with succinic semialdehyde dehydrogenase deficiency: a compound heterozygote with 103–454del and 1460T>A of the ALDH5A1 gene.. Human Heredity.

[37] Bekri S, Fossoud C, Plaza G, Guenne A, Salomons GS (2004). The molecular basis of succinic semialdehyde dehydrogenase deficiency in one family.. Molecular Genetics and Metabolism.

[38] Chambliss K, Hinson DD, Trettel F, Malaspina P, Novelletto A (1998). Two exon-skipping mutations as the molecular basis of succinic semialdehyde dehydrogenase deficiency (4-hydroxybutyric adicuria).. American journal of human genetics.

[39] Hogema B, Gupta M, Senephansiri H, Burlingame TG, Taylor M (2001). Pharmacologic rescue of lethal seizures in mice deficient in succinic semialdehyde dehdrogenase.. Nature Genetics.

[40] Malaspina P (2006). Succinic semialdehyde dehydrogenase deficiency: clinical, biochemical and molecular characterization of a new patient with severe phenotype and a novel mutation.. Clinical genetics.

[41] Struys E, Verhoeven NM, Salomons GS, Berthelot J, Vianay-Saban C (2006). D-2-hydroxyglutaric aciduria in three patients with proven SSADH deficiency: genetic coincidence or a related biochemical epiphenomenon?. Molecular Genetics and Metabolism.

[42] Akaboshi S, Hogema BM, Novelletto A, Malaspina P, Salomons GS (2003). Mutational spectrum of the succinic semialdehyde dehydrogenase (ALDH5A1) gene and functional analysis of 27 novel disease-causing mutations in patients with SSADH deficiency.. Human Mutation.

[43] Blasi P, Palmerio F, Aiello A, Rocchi M, Malaspina P (2006). SSADH variation in primates: intra- and interspecific data on a gene with a potential role in human cognitive functions.. Journal of Molecular Evolution.

[44] Lorentzen E, Hensel R, Knura T, Ahmed H, Pohl E (2004). Structural Basis of Allosteric Regulation and Substrate Specificity of the Non-Phosphorylating Glyceraldehyde 3-Phosphate Dehydrogenase from Thermoproteus tenax.. Journal of Molecular Biology.

[45] Fatemi S. H. SJM, Earle JA, Araghi-Niknam M, Eagan E (2005). GABAergic dysfunction in schizophrenia and mood disorders as reflected by decreased levels of glutamic acid decarboxylase 65 and 67 kDa and Reelin proteins in cerebellum.. Schizophrenia Research.

[46] Law R, Irving JA, Buckle AM, Ruzyla K, Buzza M (2005). The High Resolution Crystal Structure of the Human Tumor Suppressor Maspin Reveals a Novel Conformational Switch in the G-helix.. Journal of Biological Chemistry.

[47] Leslie A, Joint CCP4 + ESF-EAMCB (1992). Newsletter on protein crystallography.

[48] Collaborative Computational Project Number 4 C (1994). The CCP4 suite: programs for protein crystallography Acta crystallographica.

[49] McCoy A, Grosse-Kunstleve RW, Storoni LC, Read RJ (2005). Likelihood-enhanced fast translation functions.. Acta crystallographica.

[50] Jaroszewski L, Rychlewski L, Li Z, Li W, Godzik A (2005). FFAS03: a server for profile-profile sequence alignments.. Nucleic Acids Research.

[51] Tsybovsky Y, Donato H, Krupenko NI, Davies C, Krupenko SA (2007). Crystal Structures of the Carboxyl Terminal Domain of Rat 10-Formyltetrahydrofolate Dehydrogenase: Implications for the Catalytic Mechanism of Aldehyde Dehydrogenases.. Biochemistry.

[52] Canutescu A, Shelenkov AA, Dunbrack RL (2003). A graph-theory algorithm for rapid protein side-chain prediction.. Protein Science.

[53] Murshudov G, Vagin AA, Dodson EJ (1997). Refinement of Macromolecular Structures by the Maximum-Likelihood Method.. Acta crystallographica.

[54] Emsley P, Cowtan K (2004). Coot: model building tools for molecular graphics.. Acta crystallographica Section D, Biological crystallography.

[55] Morris R, Perrakis A, Lamzin VS (2003). ARP/wARP and automatic interpretation of protein electron density maps.. Methods in enzymology.

[56] Terwilliger T, Klei H, Adams PD, Moriarty NW, Cohn JD (2006). Automated ligand fitting by core-fragment fitting and extension into density.. Acta crystallographica.

[57] Terwilliger T, Adams PD, Moriarty NW, Cohn JD (2007). Ligand identification using electron-density map correlations.. Acta crystallographica.

[58] Davis I, Leaver-Fay A, Chen VB, Block JN, Kapral GJ (2007). MolProbity: all-atom contacts and structure validation for proteins and nucleic acids.. Nucleic acids research.

[59] Androulakis S, Schmidberger J, Bate MA, DeGori R, Beitz A (2008). Federated repositories of X-ray diffraction images.. Acta Crystallographica Section D.

[60] Hogema B, Akaboshi S, Taylor M, Salomons GS, Jakobs C (2001). Prenatal diagnosis of succinic semialdehyde dehydrogenase deficiency: increased accuracy employing DNA enzyme, and metabolite analyses.. Molecular Genetics and Metabolism.

[61] Trettel F, Malaspina P, Jodice C, Novelletto A, Slaughter CA (1996). Human succinic semialdehyde dehydrogenase: molecular cloning and chromosomal localization.. Adv Exp Med Biol.

