# Supplementary figures and images for: The X-Ray Crystal Structure of Escherichia coli Succinic Semialdehyde Dehydrogenase; Structural Insights into NADP+/Enzyme Interactions
Source: PLoS One. 2010 Feb 18;5(2):e9280. doi: 10.1371/journal.pone.0009280 (PMC2823781; doi:10.1371/journal.pone.0009280)

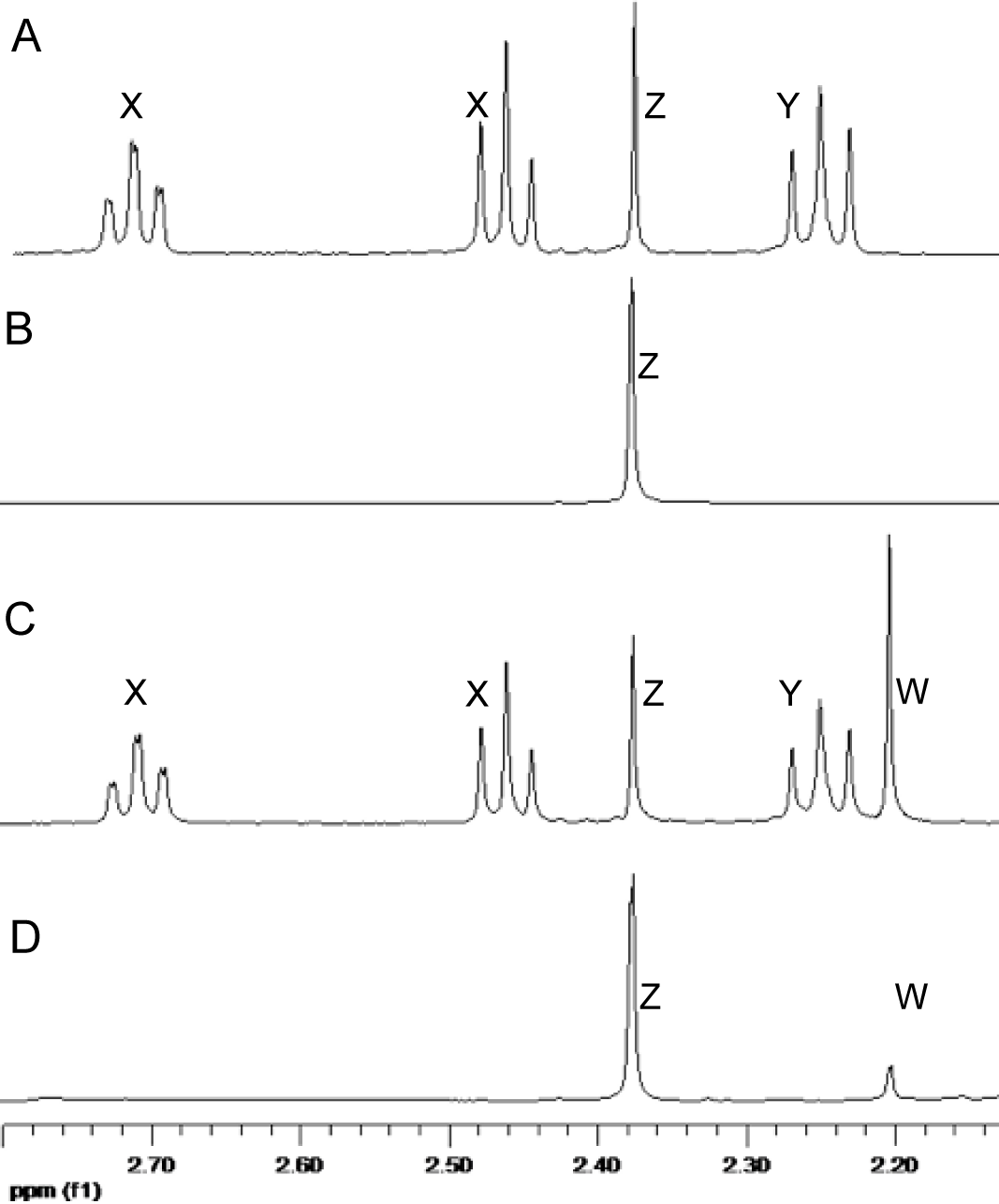

Supplement: Figure S1 — The conversion of SSA to SA using 1H NMR spectroscopy. NMR spectra of (a) substrate alone in phosphate buffer which contains succinic semialdehyde (X), 4,4-dihydroxybutanoic acid (Y) and succinic acid (Z). (b) Succinic acid alone with phosphate buffer, showing only the singlet peak of succinic acid (Z). For SSADH enzyme assay, incubation of succinic semialdehyde and SSADH in the presence of NADP+ at 0 min (c) and 240 min (d) showing all substrates (X, Y, Z) has been converted to succinic acid (Z), NADP+ is marked with W. (0.21 MB TIF) [file pone.0009280.s001.tif]

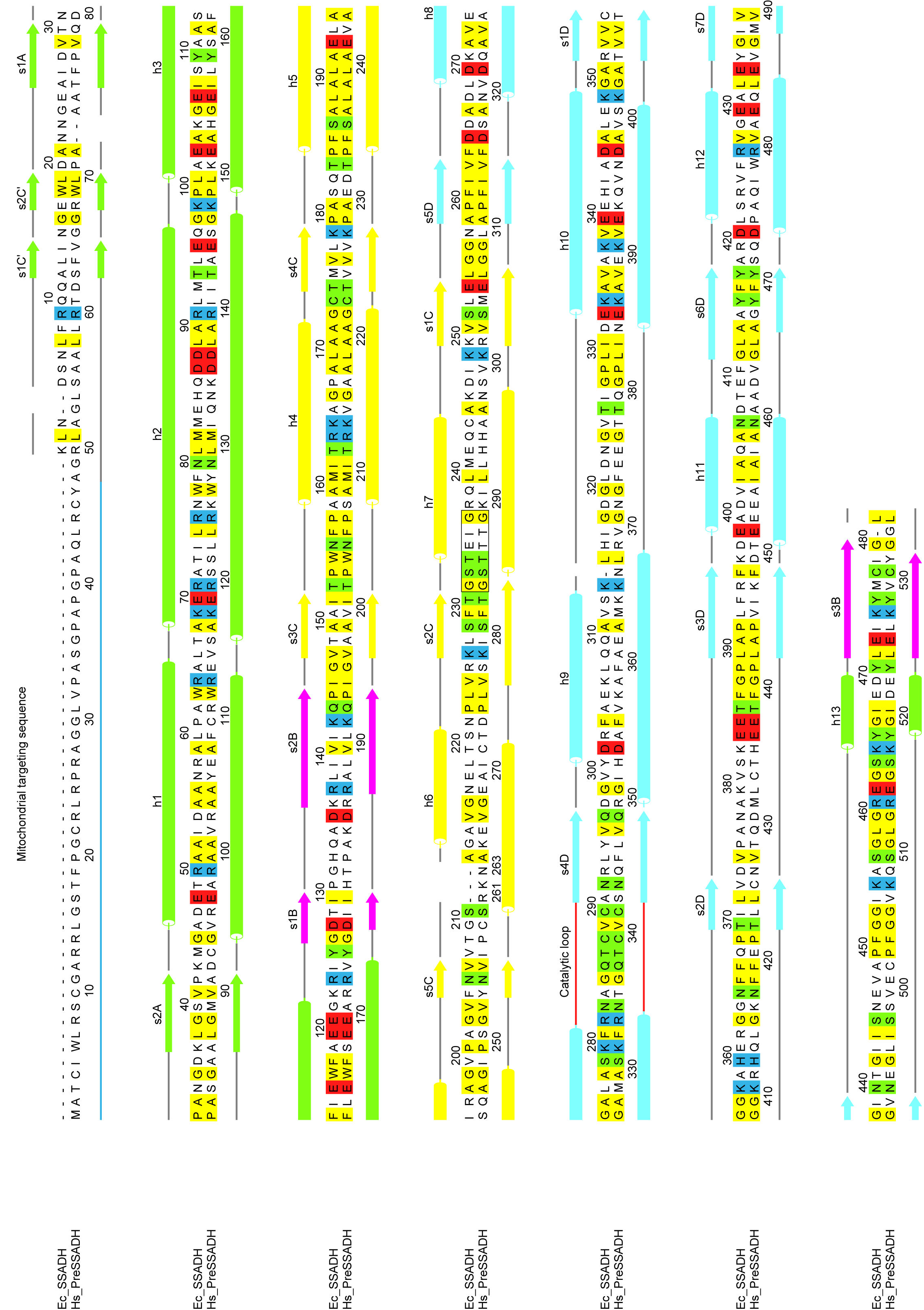

Supplement: Figure S2 — Alignment of E. coli SSADH with human SSADH. Conserved residues have been highlighted according to the following, polar (green), non-polar (yellow), acidic (red) and basic (blue). The secondary structure (E. coli SSADH above the sequence and human below the sequence) has been marked with either an arrow designating a Î2-sheet or a cylinder representing an Î±-helix. The secondary structure elements are coloured according the Figure 2a. Structurally important regions have also been marked and labelled, catalytic loop (red line) and the GXXXXG motif (box) from the Rossmann fold. The human SSADH mitochondrial targeting sequence is labelled and shown as a blue line. (1.46 MB TIF) [file pone.0009280.s002.tif]

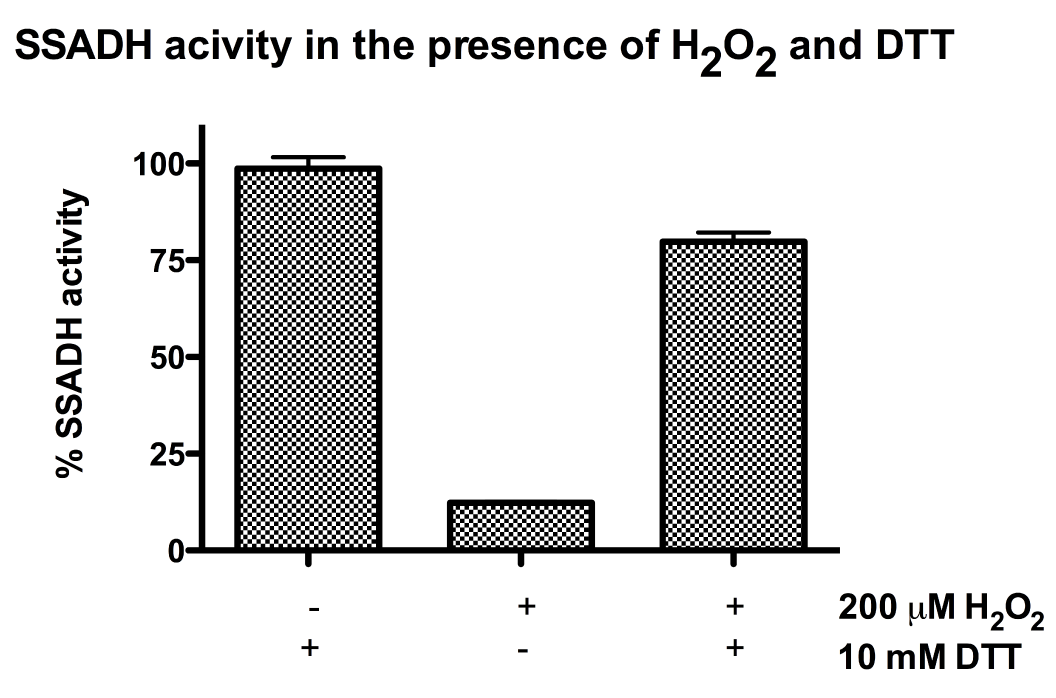

Supplement: Figure S3 — SSADH activity in an oxidised and reduced environment. The first column shows the untreated activity of SSADH. In the second column the E. coli SSADH enzyme was oxidised by incubating it with 200 Î¼M H2O, which has 11–13% of untreated SSADH activity. In the third column the addition of 10 mM DTT to previously oxidised enzyme, which rescued the inhibition to 80% that of the normal activity. (0.16 MB TIF) [file pone.0009280.s003.tif]

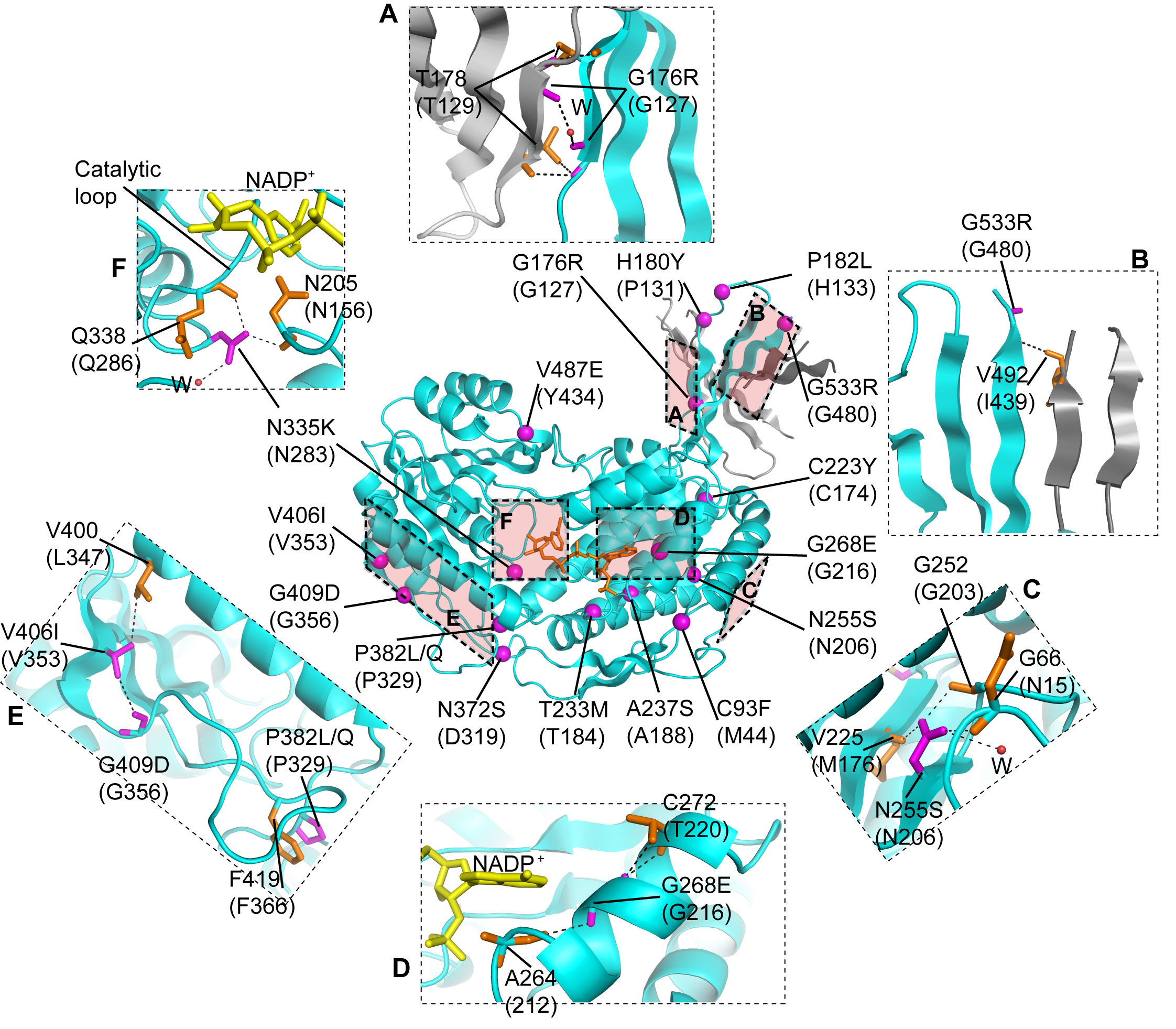

Supplement: Figure S4 — A–F. Human point mutations causing a dramatic loss of acticvity (≤2% residual SSADH) mapped onto the E. coli SSADH structure. A cartoon representation of E. coli SSADH monomer A (cyan, NADP+ orange) showing the 17 point mutations (magenta spheres) that map to the mature human protein. A small region of monomer B catalytic domain (dark grey) and monomer C oligomerisation domain (light grey) have been included to illustrate the proximity of point mutations with regard to dimer and tetramer interfaces. Labelled, dashed red boxes highlight the area of E. coli SSADH that have been enlarged for analysis. A–F) Show the equivalent E. coli SSADH residues (magenta sticks) to the human point mutants and their interactions (black dashed lines) within their immediate surrounds with other residues (orange sticks). A–B) Mutations in this region would be anticipated to disrupt dimer and tetramerisation, while C) we would anticipate decreased stability in this region due to loss of stabilising interactions with the N255S mutation. D) A loss of NAD/P+ binding efficiency would be expected with the addition of a large negatively charged residue, G268E, into the positively charged adenosine-ribose binding pocket of SSADH. E) We anticipate an overall loss of structural integrity of this loop region from either of the mutations G409D (loss of +/+ backbone conformation) and P382L/Q (disruption of aromatic ring stacking interactions with F419). F) The introduction of a large charged residue (N335K) in a buried surface on the catalytic loop would be anticipated to greatly disrupt the structural integrity and catalytic ability of SSADH. (2.71 MB TIF) [file pone.0009280.s004.tif]
