# Supplementary material for: The X-Ray Crystal Structure of Escherichia coli Succinic Semialdehyde Dehydrogenase; Structural Insights into NADP+/Enzyme Interactions
Source: PLoS One. 2010 Feb 18;5(2):e9280. doi: 10.1371/journal.pone.0009280 (PMC2823781; doi:10.1371/journal.pone.0009280)
Supplement: Table S1 — Intermolecular contacts with respect to monomer A. (0.18 MB DOC) [file pone.0009280.s005.doc]

##### Supplementary Table 1: Intermolecular contacts with respect to monomer A.

| Monomer | Residue | Atom | H = Hydrogen bond S = Salt bridge | Residue monomer A | Atom monomer A |
| --- | --- | --- | --- | --- | --- |
| B (dimerisation) | D128 | OD1 | HS | K465 | NZ |
|  |  | OD2 | H | Y466 | OH |
|  |  |  | S | K465 | NZ |
|  | Q144 | NE2 | H | E430 | O |
|  | R226 | NH1 | HS | E433 | OE1 |
|  |  |  | S | E433 | OE2 |
|  |  | NH2 | S | E433 | OE1 |
|  | R238 | NE | H | K246 | O |
|  | K246 | O | H | R238 | NE |
|  | G429 | O | H | K475 | NZ |
|  | E430 | O | H | K475 | NZ |
|  |  |  | H | Q144 | NE2 |
|  | L432 | O | H | K475 | NZ |
|  | E433 | OE1 | HS | R226 | NH1 |
|  |  |  | S | R226 | NH2 |
|  |  | OE2 | S | R226 | NH1 |
|  | G435 | O | H | Y476 | N |
|  | V437 | O | H | C478 | N |
|  |  | N | H | Y476 | O |
|  | I439 | O | H | G480 | N |
|  |  | N | H | C478 | O |
|  | I444 | O | H | Y476 | OH |
|  | N446 | ND2 | H | Y476 | OH |
|  | R461 | NH1 | S | E473 | OE1 |
|  |  | NH2 | HS | E473 | OE1 |
|  |  |  | H | I474 | O |
|  | K465 | NZ | HS | D128 | OD1 |
|  |  |  | S | D128 | OD2 |
|  | Y466 | OH | H | D128 | OD2 |
|  | E473 | OE1 | HS | R461 | NH2 |
|  | I474 | O | H | R461 | NH2 |
|  | K475 | NZ | H | G429 | O |
|  |  |  | H | E430 | O |
|  |  |  | H | L432 | O |
|  | Y476 | N | H | G435 | O |
|  |  | OH | H | I444 | O |
|  |  |  | H | N466 | ND2 |
|  |  | O | H | V437 | N |
|  | C478 | N | H | V437 | O |
|  |  | O | H | I439 | N |
|  | G480 | N | H | I439 | O |
| C (Tetramerisation) | G127 | N | H | T129 | O |
|  |  | O | H | T129 | N |
|  | T129 | O | H | G127 | N |
|  | R420 | O | H | L422 | N |
|  | L422 | N | H | R420 | O |
| D  (Tetramerisation) | K69 | NZ | S | E447 | OE1 |
|  |  |  | HS | E447 | OE2 |
|  | R76 | NH2 | S | E120 | OE2 |
|  | W117 | O | H | R124 | NH2 |
|  | E120 | OE1 | HS | R124 | NH1 |
|  |  |  | S | R124 | NH2 |
|  |  |  | HS | K123 | NZ |
|  |  | OE2 | HS | K123 | NZ |
|  |  |  | S | R76 | NH2 |
|  | E121 | OE1 | HS | R124 | NE |
|  |  |  | HS | R124 | NH2 |
|  | K123 | NZ | HS | E120 | OE1 |
|  |  |  | HS | E120 | OE2 |
|  | R124 | NH1 | HS | E120 | OE1 |
|  |  | NH2 | HS | E121 | OE |
|  |  |  | S | E120 | OE1 |
|  |  |  | H | W117 | O |
|  |  | NE | HS | E121 | OE1 |
|  | R138 | NH2 | HS | E430 | OE1 |
|  |  |  | S | E430 | OE2 |
|  |  | NE | HS | E430 | OE2 |
|  |  |  | S | E430 | OE1 |
|  | E430 | OE1 | HS | R138 | NH2 |
|  |  |  | S | R138 | NE |
|  |  | OE2 | HS | R138 | NE |
|  |  |  | S | R138 | NH2 |
|  | E447 | OE1 | S | K69 | NZ |
|  |  | OE2 | HS | K69 | NZ |

H = hydrogen bond, S = salt bridge.
