# Supplementary material for: The X-Ray Crystal Structure of Escherichia coli Succinic Semialdehyde Dehydrogenase; Structural Insights into NADP+/Enzyme Interactions
Source: PLoS One. 2010 Feb 18;5(2):e9280. doi: 10.1371/journal.pone.0009280 (PMC2823781; doi:10.1371/journal.pone.0009280)
Supplement: Table S2 — Residues involved in significant NADP+ binding using Molprobity [58]. (0.07 MB DOC) [file pone.0009280.s006.doc]

**Supplementary Table 2**: **Residues involved in significant NADP^+^ binding using Molprobity** [58].

| Residue | Monomer | Atom | Type of bond | NADP^+^ atom |
| --- | --- | --- | --- | --- |
| **T153** | A & B | O | WMHB | AO2* AO3* |
| **W155** | All | NE1 | HB | NO1 |
| **N156** | A & D | ND2 | WMHB | NN7 |
| **F157** | B | CE2 | WMHB | NN7 |
|  | B | CD2 | WMHB | NN7 |
| **K179** | All | NZ | HB | 3AOP |
|  | All | NZ | HB | AO2* |
|  | A | NZ | WMHB | AO2* & AO3* |
| N217 | A, C & D | OD1 | WMHB | AN1 & AN6 |
| **G232** | B, C, & D | O | vdw | NO7 |
| **S233** | All | OG | HB | AO2 |
|  | All | N | HB | AO2 |
| Q239 | A | OE1 | WMHB | AN6 |
| Q243 | A, C & D | NE2 | WMHB | AN1 & AN6 |
|  | A | OE1 | WMHB | AN6 |
| **E254** | A & B | OE1 | WMHB | NO7 |
| **L255** | A & B | O | vdw | NO7 |
|  | A & B | O | WMHB | NO7 |
| **C288** | A & B | SG | WMHB | NO7 & NN7 |
|  |  | N | WMHB | NN7 |
| **K338** | All | NZ | HB | NO3* |
| **E385** | All | OE1 | HB | NO2* |

HB = Hydrogen bond, WMHB = water mediated hydrogen bond, vdw = van der Waal contact. Residues in bold are conserved in human SSADH
